# News and Notes

**Published:** 1910-12

**Authors:** 


### NEWS AND NOTES

### JANUARY MEETING OF CHATTAHOOCHEE VALLEY MEDICAL ASSOCIATION. JAN 10-11.

### FIRST DAY. MORNING SESSION.

Call to order, 10:00 A. M.

Invocation, Rev. J. R. Christie, Colmubus, Ga.

Address of Welcome by Hon. Rhodes Brown, Mayor of Columbus, Ga.

Address of Welcome Behalf Muscogee County Medical Society and Physicians of Columbus, Ga., by H. Stokes Munroe, M. D., Columbus, Ga.

Response in behalf Chattahoochee Medical and Surgical Association, by Geo. M. Niles, M. D., Atlanta, Ga.

Annual Address of the President.

### AFTERNOON SESSION—2 P. M.

1. Diabetes, by C. S. Yarbrough, M. D., Auburn, Ala. Leaders in discussion, H. T. Hamner, J. M. Poer.

2. Optimistic Therapeutics—By Geo. M. Niles, M. D., Atlanta, Ga. Leaders in discussion: B. L. Wyman, A. L. Harlan.

3. Vaccine Therapy, with Special So-Called Surgical Tuberculosis—By L. M. Gaines, M. D., Atlanta, Ga. Leaders in discussion, H. P. Cole, Jas. C. McLester.

4. Puerperal Sepsis—from a general practitioner's standpoint.—By H. S. Bruce, M. D, Opelika, Ala. Leaders in discussion: J. A. Goggans, J. H. McDuffie.

5. Some uses of Normal Saline Solution.—By V. H. Williams, M. D., West Point, Ga. Leaders in discussion: H. P. Cole, C. A. Dexter.

6. Cerebral Tumor, with report of a case.—By L. W. Johnston, M. D., Tuskegee, Ala. Leaders in discussion: Jno. D. S. Davis, L. L. Hill.

7. Some thoughts on the management of Typhoid Fever. By W. W. Stevenson, M. D., Roanoke, Ala. Leaders in discussion: C. L. Williams, J. J. Winn.

8. Dysmenorrhea.—By J. R. B. Branch, M. D., Macon, Ga. Leaders in discussion: R. S. Hill, Gaston Torrence.

9. Review of Fifty-Two Consecutive Operations for fibroid tumors in the Uterus.—By Edward G. Jones, M. D., Atlanta, Ga.. Leaders in discussion. Gaston Torrence, R. S. Hill, J. A. Goggans.

10. A consideration of the Brain in reference to intellectuality.—By W. H. Hudson, M. D., Atlanta, Ga. Leaders in discussion; B. L. Wyman, James L. Cambell.

11. Habitual Constipation.—By G. M. Saliba, M. D., Roanoke. Leaders in discussion: Geo. M. Niles, J. M. Welch, H. R. Slack.

12. Fracture of the Clavicle.—By W. D. Gaines, M. D., LaFayette, Ala. Leaders in discussion: E. P. Green, Hugh McCullough, W. L. Cooke.

### SECOND DAY.

### MORNING SESSION—8:30 A. M.

13. Conservative Surgery of the Ovary.—By F. K. Boland, M. D., Atlanta, Ga. Leaders in discussion: H. S. Munroe. R. S. Hill, H. B. Disharoon.

14. Rheumatism.—By W. G. Floyd, M. D., Roanoke, Ala. Leaders in discussion: A. B. Bennett, E. P. Green, T. H. Haralson.

15. Chronic Chloecystitis.—By Jas. L. Campbell, M. D., Atlanta, Ga. Leaders in discussion: H. T. Hamner, H. S. Bruce, Velpeau Langley.

16. Ectopic Cestation.—By R. S. Hill, M. D., Montgomery, Ala. Leaders in discussion: J. R. B. Branch, E. G. Jones, L. W. Johnston.

17. Phrophylaxis in Surgery.—By Jno. D. S. Davis, M. D., Birmingham, Ala. Leaders in discussion, J. S. Campbell, L. M. Gaines, W. T. Langley.

18. Report of Twenty Cases of blood transfusion.—By H. P. Cole, M. D., Mobile, Ala. Leaders in discussion. Jno. D. S. Davis, W. L. Cooke, Thos. F. Taylor.

19. Cerebral Hemorrhage affecting the Speech Center.—By J. L. Bowman, M. D., Union Springs, Ala. Leaders in discussion: Gaston Torrence, Jas. L. Campbell.

20. Surgery of Ectopic Gestation.—By Gaston Torrence, M. D., Birmingham, Ala. Leaders in discussion: R. S. Hill, J. G. Palmer, J. A. Goggans.

21. Local Anesthesia.—By W. L. Cooke, M D,, Columbus, Ga. Leaders in discussion: W. W. Stevenson, H. S. Persons, H. S. Munroe.

22. Pneumonia.—By R. T Dorsey, M. D., Atlanta, Ga. Leaders in discussion: J. M. Welch, L. M. Gaines, W. F. Dickinson, H. W. Terrell.

23. Some effects of a lost art of the Tongue.—By M. M. Stapler, M. D., Macon, Ga. Leaders in discussion: Geo. W. Chambers, Hugh M. Lokey, W. L. Bullard.

24. Diagnosis and management of Tuberculosis from the Standard of a General Practitioner.—J. P. Motley, M. D., Wadley, Ala. Leaders in discussion: L. M. Gaines, C. S. Yarborough, C. A. Dexter.

AFTERNOON SESSION, WEDNESDAY—2 P. M.

25. Starvation as cause of digestive disturbances.—By Jas.

S. McLester, Birmingham, Ala. Leaders in discussion: Geo. M. Niles, Williams Wade Harper.

26. Indigestion in Childhood.—By C. G. Laslie, M. D., Montgomery, Ala. Leaders in discussion: Jas. S. McLester, Velpeau Langley.

27. Account of visit to Berlin Clinics and a special study of the Ehrlich-Hata Remedy 606.—By E. G. Ballanger, M. D., Atlanta, Ga. Leaders in discussion: Jas. S. McLester, L. M. Gaines.

28. Feeding in infantile Gastro-intestinal indigestion.—By William Wade Harper, M. D., Selma, Ala. Leaders in discussion: C. G. Leslie, Velpeau Langley, L. M. Gaines.

29. The Thyroid Gland; its diseases and treatment.—By L. C. Fischer, M. D., Atlanta, Ga. Leaders in discussion: F. M. Ridley, Sr., A. L. Harlan.

30. The Significance of Ascites.—By W. P. Dickinson, M. D., LaFayette, Ala. Leaders in discussion: H. P. Disharoon, Hugh McCulloh.

31. The importance of the Eradication of Hook Worm.—By H. G. Perry, M. D., Greensboro, Ala. Leaders in discussion: W. H. McClendon, H. R. Slack.

32. Entero Collitis in infants and young children.—By E. V. Langley, M. D., Camp Hill, Ala. Leaders in discussion: Wm. Wade Harper, C. G. Laslie.

33. The significance of Headache and its treatment by the general practitioner.—By Charles O. Williams, M. D., West Point, Ga. Leaders in discussion: G. M. Saliba, E. H. Sims.

34 Report of a case of Cardia Spasm.—By C. A. Dexter, M. D., Columbus, Ga. Leaders in discussion: J. M. Welch, H. W. Terrell.

35. A new Method of Treating the Opium Habit.—By B. W. Allen, M. D., Columbus, Ga. Leaders in the discussion: A. B. Bennett, C. S. Yarbrough.

36 Some remarks on Infantile Convulsions.—By T. C. Hodge, Atlanta, Ga. Leaders in discussion: Wm. Wade Harper, J. P. Motley.

37. Non-surgical treatment of Uterine Flexion.—By W. H. Moon, M. D., Goodwater, Ala. Leaders in discussion: S. H. Newman, O. M. Steadham.

### REGULAR MEETING OF FULTON COUNTY MEDICAL SOCIETY, OCTOBER 20, 1910.

REPORTED BY R. R. DALY, M. D.

Dr. R. R. Kime read a paper on "Gynecologic Perineal Repair."

Dr. Archibald Smith said that there were a few points in which he differed from the Doctor's method. Denudations should be made to lessen the cicatricial formation. The edges of the levator ani should be well brought up into the wound in fresh cases, thus establishing better support to the perineum. This is also true in second repair and it is far better than simply bringing the edges together under the fascia. The question of surutes has been decided against the chromic gut but plain gut sometimes is not strong enough enough. Kangaroo tendon is often better.

Dr. Earnest said that the drawing did not seem to represent a complete tear; in complete tear the union of the perineal muscles is of first importance. Some modification of the Emmett operation has been successful in his hands. He repairs the mucous membrane of the rectum first and then proceeds as in the Emmett. Buried simple gut is used except in the outside sutures where silver wire and shot are applied if needed. He commends Dr. Kime's paper as a whole.

Dr. Ellis said he appreciated Dr. Smith's criticism as to getting rid of cicatrical tissues. Dr. Kime's method is excellent in that no tissue is lost if a secondary operation has to be done.

Dr. Kime said in closing that the scar tissues are to be cut out in his work. The flap-splitting method he has used for over fifteen years, carefully building up the perineum and has had no failures. He does not like the Prestine flap. As to bringing together the levatores ani, he cannot dissect any of these muscles under operative conditions and he does not want to do it on a live subject because of the bleeding. Therefore, if the ends of the muscles are brought together in the bite of the needle, they will give good union because they are raw surfaces and not muscles side by side. The fibres of the various muscles decussate anyway in the perineal body. He quoted extensively from Cornell anatomists in support of the flap-splitting method.

Dr. Dunbar Roy read a paper on "Chronic Nasal Pharyngeal Bursitis."

Dr. Lokey said he hoped he would get something more on the line of treatment because the literature on this subject is so unsatisfctory. He had a case with a median fissure adenoid removed with an adenoid curette but the fissure remained. 10% nitrate of silver solution gave him better results than anything else.

Dr. Daly said that he had a case in the Tuberculosis clinic giving a typical effect in scab of the naso pharynx with pocket fissure in the adenoid underneath that resisted all ordinary treatment. As Dr. Roy had pointed out, the naso pharynx was very tolerant of manipulations. All the glandular tissue was removed with careful curettage and the parts subsequently rubbed with the finger tip at repeated intervals. In addition nitrate of silver, 20 grs. to the ounce, was applied, and recovery followed.

Dr. Roy said in closing, that this condition was distinctly different from ordinary post-nasal catarrh and must be studied from its scab symptoms to its anatomical condition. Then the treatment that would seem most likely to be of avail was surgical.

---

### PROCEEDINGS OF THE AMERICAN PROCTOLOGICAL SOCIETY.

*(Continued.)*

---

### "SIGNIFICANCE OF RECTAL HEMORRHAGE."

By Louis J. Krouse, M. D., of Cincinnati, Ohio.

---

Who called the attention of the profession to the importance of making a more careful examination of every case where there is bleeding from the rectum. He stated that rectal hemorrhage must not be considered conclusive of the existence of piles. Many other diseases besides piles are accompanied with bleeding. He laid great stress on the importance of diagnosing malignancy in its early stage so as to give the patient a better chance of recovery. Many cases of malignant disease of the rectum, whose only symptom is hemorrhage have been overlooked and the patient sacrificed which would not have occurred had the family physician insisted upon a local examination thereby diagnosing the disease in its incipiency before it had gone beyond the operable stage. He further stated that every patient is entitled to a thorough examination; and physicians are in duty bound to use all the means at their command to accomplish it. As Murray very aptly expressed himself "Thus a case that today would be operable and a cure result, if diagnosed, would be inoperable in six months or a year and death result." The author reported numerous cases where a correct diagnosis had not been made on account of the negligence of the family physician. Some had been operated upon for bleeding piles which subsequently turned out to be cancer. He concluded his article with the statement "that earlier recognition of malignancy would add materially to the future welfare of the patient which can be obtained by surgical measures, and it therefore, behooves the general practitioner to be on his guard and examine carefully every case of bleeding so as to detect malignancy in its incipient stage."

---

### "ANO-RECTAL AFFECTIONS OF INFANCY AND CHILDHOOD."

By A. J. Zobel, M. D., San Francisco, Cal.

This paper briefly described those ano-rectal affections of infancy and child-hood which may appear on one's daily work or in consultation practice.

From the first hour after birth the ano-rectal regoin is of vast importance. At that time malformations may be determined and proper relief promptly afforded.

The various malformations were enumerated and briefly described. Some of these abnormalities pass unnoticed throughout a long life but others are the source of great discomfort and distress.

Mention was made that while hemorrhoids are common in adults the posibility of their presence in the young is rarely considered. Yet they may appear in children of tender years. The various causes for hemorrhoids in the young were reviewed in this paper.

Malignant growths of the rectum while rare are occasionally met with. Cases were quoted where the disease was found in children as young as five years of age.

Benign growths are more common. Adenoma is the most frequent of these. They are often diagnosed as internal hemorrhoids, and like them, may become strangulated. They may exist for some time and attain quite a size without producing any symptoms until strangulation occurs.

Fissure of the anus is believed by the writer to be present more often than it is usually diagnosed. It may cause severe cyring in nurslings. May cause reflex symptoms to appear which for a time may baffle the diagnostician. Some of these may resemble coxalgia. The incautious and improper introduction of syringe nozzles and thermometers into the anal canal frequently cause fissures. Other causese were also mentioned.

Especial stress was laid on the subject of Pruritus Ani in children. The writer believing it to be a very frequent source of great discombort and torment to the little ones. It is very rarely suspected or diagnosed and he believes that it it accounts for much of that peevishness in these little ones for which no cause can usually be assigned. The child is seen to rub his anal region saying, "It hurts." Does not complain of itching. Seems to misinterpret the sensation. He has found superficial lesions of the anal mucous membrane in these cases, and as the symptoms disappeared when local treatment was instituted he feels assured that these were the cause of the trouble.

Fistulo-in-ano is met with occasionally in children and even in nurslings. While it may be tubercular it may also be of a congenital nature.

Ischio-rectal abscesses are met with even in early infancy. When incised they rarely end in fistulae.

Prolapse of the mucous membrane of the anus and rectum is a common condition during the second and third years of life. Lony continued tight binding in babyhood may bet he starting point. Diarrhea is the most common antecedent. Anything that induces prolonged and severe straining at stool may be a cause. Some of these causes were mentioned.

The varities and causese of proctitis were also dwelt upon. Proctitis is often taken for ordinary catarrhal diarrhae due to improper feeding. It is advised that when a gonorrhea of the genital tract exists in children that a secondary infection of the ano-rectal region should always be considered.

It is hoped that this reminder that infants and children have ano-rectal troubles, as well as adults, will lead to more thought being given in this direction, and that it will bear fruit in bringing relief to some of these little sufferers.

---

### "THE TREATMENT OF RECTAL FISTULA."

By J. Rawson Pennington, M. D., of Chicago, Ill.

Who referred to three methods, viz.; simple incision; the injection of bismuth paste; the incision or excision with immediate suture, (Proctorrhaphy.)

Of the Simple Incision he said: Those of us who are operating quite frequently for this malady know its disadvantage, drawbacks, and frequent failures to cure. That this operation has done more than any other, unless it be that of the ligature or clamp and cautery operation for hemorrhoids, to bring disrepute upon the rectal surgery. That the laity dread a rectal operation more than any other surgical procedure because of the fear of pain, the fear of recovery and the fear of loss of control over the bowels. Yet, we know that each of the above operations in the hands of experts give good results. Concerning the injection of bismuth paste, he said: To treat a rectal fistula, the paste is liquefied by heating in a water-bath and injected into one of the openings with a metal or glass syringe. The other opening or openings are kept closed by an assistant while the injection is being made. Enough force is used until one feels reasonably sure that all tracts and diverticuli have been filled. The paste may be forced into some line of cleavage if too much tension is used and carried along this line to some distant organ or healthy tissue and deposited there with deleterious results.

Of excision or incision with immediate suture, (Proctorrhaphy) he said: This method is the most rational of all surgical procedures, that he dissects and removes the entire tract when a probe or director can be passed through the fistulous channel and into the rectum. That he then searches out and removes any diverticuli or tracts connected with the main tract. If this can not be, or should not be done he then incises the fistula and dissects out all granulation tissue. If needs be the wound is disinfected with carbolic acid and alcohol.

Suturing the wound may be done by lembertizing the line of incision from its termination in the rectum to the anus. The ends of the severed sphincters as well as the deeper portions of the incision are next brought together with interrupted catgut sutures. The skin and fescia are sutured with interrupted silk worm-gut. He dresses the wound with iodoform or plain gauze and applies a T bandage. He maintains that Proctorrhaphy, or the paste, or a combination of the two, offers the nearest approach we have to the ideal method of treating extensive rectal fistula.

---

### "THE TUBERCULIN REACTION IN CASES OF PERIRECTAL INFECTION."

By Collier F. Martin, M. D., of Philadelphia, Pa.

The author was so impressed with the frequent coincidence of pulmonary tuberculosis and perirectal infections that he began a series of tests and examinations to determine their relation.

He usese the Moro tuberculin reaction, combined with physical and bacteriologic examination.

In the preliminary report of 36 cases, which he divides in two groups, he got the following results.-

Group 1.—Rectal pyogenic infections, including here fistulae, abscesses, and deep rectal ulceration. There were 20 Positive reactions out of 21 cases. The negative case was one profundly tuberculous.

Group II.—Non-pyognic rectal cases. There were 11 cases, includinf hemorrhoids, fissue, and catarrhal proctitis, with three positive tuberculin reactions. This he holds, is probably the ratio of tuberculosis in this class of cases. One negative case in this group was intensely tubercular, with extensive lung lesions evident, and with abundant tubercle bacilli in the scputum.

Accepting the tuberculin test as a specific one, he got 100% positive in Group I, and about 36% in Group II. The four cases giving negative reactions, yet being proved tuberculous, by sputum examination, proved to be of very low resistance, two dying in a few months and two, at present, in a precarious condition.

He emphasizes "continued history taking" as being extremely valuable to the proper appreciation of the case.

The author places particular stress on the prognostic value of the tuberculin test.

Accepting the positive reaction to tuberculin as indicative of a tuberculous lesion somewhere in the body, his conclusions are as follows:-

1. Two consecutive, negative reactions, with no physical signs in evidence, is conclusive proof of the absence of such lesions.

2. Two consecutive negative or feeble reactions, with physical signs of a lesion somewhere, is indicative of a very grave prognosis.

3. The degree of the reaction is directly proportionate to the degree of the resistance of that individual.

4. That the tubercule bacillus, like no other, reduces the bodily defences to pyogenic invasion.

5. That in practically all rectal pyogenic infections, there is a tuberculous lesion somewhere in the body.

6. That the classification of perirectal infections into tuberculous and non-tuberculous is untenable.

His investigations have caused the author to raise the following questions;-

1. Is the primary tuberculous lesion pulmonary?

2. Is the local infection tuberculous?

3. Do the tubercule bacilli gain entrance into the body through the respiratory or the alimentary tract?

4. Is such infection carried to the rectal and perirectal tissues by the blood current, the lymphatics, or directly, by the fecal current?

5. How does the tubercle bacillus influence the pyogenic infections, locally, as in mixed infection, or by lowering the body-resistance to the invasion by pyogenic bacteria.?

---

### "LANE'S CONCEPTION OF CHRONIC CONSTIPATION AND ITS MANAGEMENT."

By A. B. Cooke, M. D., of Nashville, Tenn.

In his monograph entitled "The Operative Treatment of Chronic Constipation" Mr. Lane first defines the scope of the treatise by stating that the term, chronic constipation, as he employs it, includes all those conditions which are 'the consequences of the accumulation of material in the intestinal tract for a period sufficiently in excess of the normal to produce on the one hand alteration in the gastro-intestinal tract and in other viscera, and on the other hand, toxic changes from absorption. The fact is emphasized that while constipation is usually marked by infrequent hard tools, there may be a daily evacuation, and in exceptional cases the motions are loos and frequent.

The two chief pathologic factors in the production of chronic constipation, according to the author, are enteroptosis and acquired mesenteries or ahensions, the latter resulting not from inflammation, but being developed to oppose the displacement of viscera, the tendency to which exists whenever the erect posture of the trunk is assumed. The displacement and fixation of the several portions of the colon in faulty positions result primarily in defective drainage, and secondarily in auto-intoxication and pathologic changes both in the gut itself and in the other abdominal viscera.

After describing these changes in detail, the author proceeds to discuss their immediate and remote effects, advancing the idea that in many cases diseases of the appendix, gall-bladder, stomach, duodenum, pancreas, kidneys, ovaries, etc., must be regarded as sequellae of chronic constipation. In addition the phenomena resulting from toxic absorption are graphically described and the importance of their recognition stressed.

With reference to treatment Lane says that 'in no circumstances should operative interference be contemplated till the surgeon has satisfied himself that every means of treatment has failed, whether medical or mechanical'. The surgery indicted depends upon the conditions present. In mild cases in which non-operative measures have failed, division of the adhesions and constricting bands may be effective. Severer cases call for more radical surgery consisting either in dividing the ileum and anastomosing it with the simgoid or upper rectum, thus short-circuiting the fecal current, or, when pain is a prominent factor in the case, removal of the colon in addition.

The writer of the paper, after personal observation of Lane's work, regards his conception of the nature and management of the malady with much favor and thinks it entitled to serios consideration at the hands of the profession.

---

### "A UNIQUE CASE OF LACERATION OF THE SPHINCTER ANI."

By Dr. A. B. Cooke of Nashville, Tenn.

On February 26, 1910, the patient a boy, seven years old, was brought to him at St. Thomas Hospital, accompanied by his Father and physician. The following remarkable history was related:- About noon on the day named the boy, who lived on a farm, went out to his favorite place behind the corn-crib to attend to a call of nature. While engaged in the act, a pet dog, a hound of middle size, came up from the rear and mounting him affected entrance into the anus and became accoupled. The boy's out-cries quickly brought his Mother upon the scene. The dog had reversed his position and was in the same relation to the boy as is ordinarily assumed in the natural act with a bitch. . The Mother's excitement was naturally marked and in her frantic efforts to disentangle the two she used considerable violence and finally succeeded in seperating the dog.

The family physician on his arrival found that the hemorrhage had practically ceased, but upon inspection of the bowel found the parts were badly lacerated and advised the patient's removal to Nashville for treatment.

Dr. Cooke's examination found very little evidence of external injury. Traction upon the anus, however, showed that several lacerations of considerable extent were present. Under general anesthesia the deepest of these was found to be in the middle line posteriorly, extending from a point two inches up the rectum through the sphincter muscles, and out upon the skin surface for a distance of approximately one inch. The external sphincter was torn in two places at this site, one tear being complete, and other partial. Anterior ly there was a second laceration, into but not through the fibers of the sphincter. In addition there was a number of minor tears in the anal margin involving the superfical tissue only.

Fourteen interrupted cat-gut sutures were used in repairing the posterior laceration, and four in the anterior one. The others did not require suturing. The result was entirely satisfactory. Union was prompt and complete and the patient returned home in two weeks with perfect sphincter control.

---

### "MULTIPLE ADENOMATA."

By Geo. W. Combs, M. D., of Indianapolis, Ind.

An adenoma is the result of an increase in number and a crowding together of elongated and enlarged secreting follicles. It is an exaggeration of epithelial cells. This epithelium is prone to penetrate the basement membrane. When it does so and reaches the muscularis and other sub-mucous tissues it is malignant. Irritation causese the transformation from the benign to the malignant. This irritation may be through the normal function of the bowel, that caused by parasites, or as a result or surgical removal singly. Surgical disturbance in situ of a benign adenoma, a widening experience shows, will be followed by malignancy.

A case was reported in which occurred the malignant degeneration without surgical interference. This does not necessarily show an inherent tendency of benign adenomata to malignancy, but the adenomata, through the factor of irritation, predisposes the patient to cancer. In the case to which reference is made above, one or more of the adenomata low down in the rectum had undergone the malignant transformation. On account of the extent of involvement and the extreme exhaustion of the patient, extirpation of carcinoma was deemed inadvisable, but a left colostomy was made reaching a portion of the sigmoid above the growth limit. The tenesmus and diarrhea were at once relieved and the patient made comfortable until the carcinoma reached the cutaneous margin. Through the colostomy lavage was administered, the solutions being normal salt, Boracic Acid and Sodium Salicylate. The adenomata between the colostomy would and the carcinoma, through functional rest of the bowel and cleanliness, disappeared.

If degeneration has not taken place a colostomy right or left, high enough to get above the growth limit, is advised and through this soothing and cleaning solutions used, rather than the removal of the whole bowel promixalward above the high limit of growth. The latter is a very serious operation for the strong and one in which the mortality will necessarily run high in these patients, as they present themselves usually late in the disease.

After malignant transformation has taken place, it would seem useless to remove the malignant portion unless the entire bowel involved may be removed at the same time.

*(To be Continued.)*

### ARMY MEDICAL CORPS EXAMINATIONS.

The Surgeon General of the Army announces that the first of the preliminary examinations for the appointment of first lieutenants in the Army Medical Corps for the year 1911, will be held on January 15, 1911, at points to be hereafter designated.

Full information concerning the examination can be procured upon application to the "Surgeon General, U. S. Army, Washington, D. C." The essential requirements to securing an invitation are that the applicant shall be a citizen of the United States, shall be between twenty-two and thirty years of age, a graduate of a medical school legally authorized to confer the degree of doctor of medicine, shall be of good moral character and habits, and shall have had at least one year's hospital training or its equivalent in practice before graduation. The examinations will be held concurrently throughout the country at points where boards can be convened. Due consideration will be given to localities from which applications are received, in order to lessen the traveling expenses of applicants as much as possible.

The examination in subjects of general education (mathematics, geography, history, general literature, and Latin), may be omitted in the case of applicants holding diplomas from reputabel literary or scietnific colleges, normal schools or high schools, or graduates of medical schools which require an entrance examination satisfactory to the faculty of the Army Medical School.

In order to perfect all necessary arrangements for the examination, applications must be complete and in possession of the Adjutant General on or before anuary 2, 1911. Early attention is therefore enjoined upon all intending applicants. There are at present seventy-six vacancies in the Medical Corps of the Army.

